# Investigation of the Epithelial to Mesenchymal Transition (EMT) Process in Equine Papillomavirus-2 (EcPV-2)-Positive Penile Squamous Cell Carcinomas

**DOI:** 10.3390/ijms221910588

**Published:** 2021-09-30

**Authors:** Federico Armando, Samanta Mecocci, Virginia Orlandi, Ilaria Porcellato, Katia Cappelli, Luca Mechelli, Chiara Brachelente, Marco Pepe, Rodolfo Gialletti, Alessandro Ghelardi, Benedetta Passeri, Elisabetta Razzuoli

**Affiliations:** 1 Department of Pathology, University of Veterinary Medicine Hannover, Bünteweg 17, 30559 Hannover, Germany; 2 Department of Veterinary Medicine, University of Perugia, 06126 Perugia, Italy; samanta.mecocci@studenti.unipg.it (S.M.); ilariaporcellatodvm@gmail.com (I.P.); katia.cappelli@unipg.it (K.C.); luca.mechelli@unipg.it (L.M.); chiara.brachelente@unipg.it (C.B.); marco.pepe@unipg.it (M.P.); rodolfo.gialletti@unipg.it (R.G.); 3 Department of Veterinary Science, University of Parma, 43121 Parma, Italy; virginia.orlandi@studenti.unipr.it; 4 Centro di Ricerca sul Cavallo Sportivo, Department of Veterinary Medicine, University of Perugia, 06126 Perugia, Italy; 5 Azienda Usl Toscana Nord-Ovest, UOC Ostetricia e Ginecologia, Ospedale Apuane, 54100 Massa, Italy; 6 National Reference Center of Veterinary and Comparative Oncology (CEROVEC), 16129 Genova, Italy; elisabetta.razzuoli@izsto.it

**Keywords:** EMT, Horse, EcPV2, penile squamous cell carcinoma, wnt/βcatenin pathway, RANKL

## Abstract

Equine penile squamous cell carcinoma (epSCC) is the most frequent tumor of the external male genitalia, representing 67.5% of equine genital cancers. epSCC is associated with papilloma virus (PV) infection and has been recently proposed as a model for human PV-induced squamous cell carcinomas. It has already been suggested that epSCC might undergo epithelial-to-mesenchymal transition (EMT). This work aims to investigate in detail this process and the possible role of PV oncoproteins in epSCC. For this purpose, 18 penile SCCs were retrospectively selected and tested for both EcPV2 presence and oncoproteins (EcPV2 E6 and EcPV2 E7) expression. Moreover, immunohistochemical EMT characterization was carried out by analyzing the main epithelial markers (E-cadherin, β-catenin, and pan-cytokeratin AE3/AE1), the main mesenchymal markers (N-cadherin and vimentin), and the main EMT-related transcription factors (TWIST-1, ZEB-1). PCR analysis was positive for EcPV2 in 16 out of 18 samples. EMT was investigated in epSCC positive for EcPV2. The immunohistochemistry results suggested the presence of EMT processes in the neoplastic cells at the tumor invasive front. Moreover, the significant upregulation of *RANKL*, together with *BCATN1*, *LEF1*, and *FOSL1* genes, might suggest a canonical Wnt pathway activation, similarly to what is reported in human penile squamous cell carcinomas

## 1. Introduction

Squamous cell carcinoma (SCC) is among the most common epithelial malignant neoplasia in horses and equids and accounts for 7–37% of all skin lesions. Although SCC may potentially affect the skin and mucosa at any site, the preferred sites for its development are the non-pigmented skin and muco-cutaneous junctions, such as eyelids and external genitalia of both male and female horses [1]. Equine penile squamous cell carcinoma (epSCC) originates from the uncontrolled proliferation of squamous epithelial cells (keratinocytes) of the penile epithelial lining.

EpSCC is, significantly, the most frequent neoplasm in the equine external male genitalia, with an incidence of 67.5% [2]. It occurs mostly in older animals with a de novo onset or from the malignant transformation of a squamous papilloma, which should therefore be considered a pre-malignant lesion [3].

A proportion of epSCC appear to be induced by *Equus caballus* Papillomavirus 2 (EcPV2), [4] which plays the role of etiological agent in tumor pathogenesis. In humans, almost all cervical carcinomas, a high percentage of ano-genital SCCs, and a subgroup of head and neck SCCs are caused by high-risk human papillomavirus infection (hrHPV). Human precancerous penile lesions and SCC induced by hrHPV show immunophenotypic, cytological, and histopathological characteristics similar to those of genital papilloma and SCC in horses [5]. Moreover, in the last few years, different studies pointed out also molecular and immunological similarities. [6,7,8] These data suggest that the horse may represent a potential spontaneous model for this tumor to study the human counterpart [9].

The inappropriate reactivation of the epithelial–mesenchymal transition (EMT) program, which is essential in the physiological processes of embryogenesis [10], and of fibrosis [11] has been proposed as the critical mechanism for the acquisition of invasive malignant phenotypes by primary epithelial cancer cells during the metastatic process [12].

EMT is characterized by a prompt activation of the so-called “master genes regulators” (i.e., TWIST, ZEB, SNAIL1, SLUG), leading to decreased expression of typical epithelial proteins (i.e., E-cadherin and cytokeratin) and promoting the gradual acquisition of a mesenchymal phenotype [13]. One of the most characteristic events in cells undergoing EMT is the so-called “cadherin switching”, characterized by a decreased E-cadherin and a transiently increased N-cadherin expression by the tumor [14,15,16]. In addition, it has been reported that the canonical wnt/β-catenin pathway activation could trigger the EMT process in equine genital squamous cell carcinomas [6].

Moreover, in some HPV-induced carcinomas, viral oncoproteins (E6 and E7) have been shown to play a major role in inducing EMT [17,18]. Therefore, based on our previous findings in an equine laryngeal squamous cell carcinoma expressing the E6 oncoprotein and undergoing EMT [19], it is hypothesized that in epSCC induced by EcPV-2, an association between viral oncoproteins expression and the triggering of EMT can exist.

This study reports for the first time an in-depth investigation of EMT markers in 15 EcPV-2-associated epSCC and 5 penile mucosa non-pathological samples. The main epithelial (E-cadherin, β-catenin, and cytokeratin) and mesenchymal (N-cadherin and vimentin) markers and the main transcription factors (TWIST-1 and ZEB-1) associated with EMT were analyzed by immunohistochemistry; genes involved in the wnt/β-catenin pathway were studied by RT-qPCR.

Based on our previous findings [6,19], the aim of this study was to confirm that EcPV-2-associated epSCC might undergo the EMT process and that this is most likely associated with the activation of the canonical wnt/β-catenin pathway and EcPV2 oncogenes expression. These findings support future studies in the equine species as a promising spontaneous animal model for human genital squamous cell carcinomas reported to lose E-cadherin expression and neo-express vimentin while undergoing the EMT process [20].

## 2. Results

### 2.1. Case Selection, EcPV2 Detection, and EcPV2 Oncogenes Expression

Eighteen cases of equine penile squamous cell carcinomas (epSCC) meeting the inclusion criteria were retrospectively selected. Results from a histopathological revision of the cases confirmed the original diagnosis of invasive SCC, whereas in one case, the diagnosis was of SCC in situ (CIS). Only one case showed morphological evidence of viral cytopathic effects (koilocytosis). Moreover, the five samples of clinically healthy penile mucosa were confirmed to be without any pathological lesions.

The B-2-microglobulin (*B2M*) gene was amplified in all samples under study, which were therefore considered suitable for the investigation of the *E6* and *E7* genes. Interestingly, 16 out of 18 samples (88.9%) showed the presence of EcPV2 E6 and E7 DNA. Among these, 12 cases (75%) expressed *E6* at the mRNA level and 11 (68.8%) expressed both E6 and E7 oncogenes (Table 1). In addition, samples from non-pathological penile mucosa resulted to be all amplifiable and negative for EcPV2 E6 and E7 detection.

Based on these results, the two negative samples for EcPV2 and the sample that resulted to be a carcinoma in situ (CIS), were excluded from the further analysis. Considered that almost all the other 15 cases were found to express either E6 or E6 and E7, we investigated whether these samples were undergoing the EMT process, compared to the non-pathological and EcPV2-free five samples (control group).

### 2.2. Intermediate Filaments Rearrangements and Cadherin Switching Suggest the Activation of the EMT Process in EcPV2-Associated Equine Penile Squamous Cell Carcinomas

In order to assess whether EcPV-2-associated epSCC undergo the EMT process, 15 EcPV2 positive epSCC were investigated for epithelial and mesenchymal markers expression.

**Figure 1:**
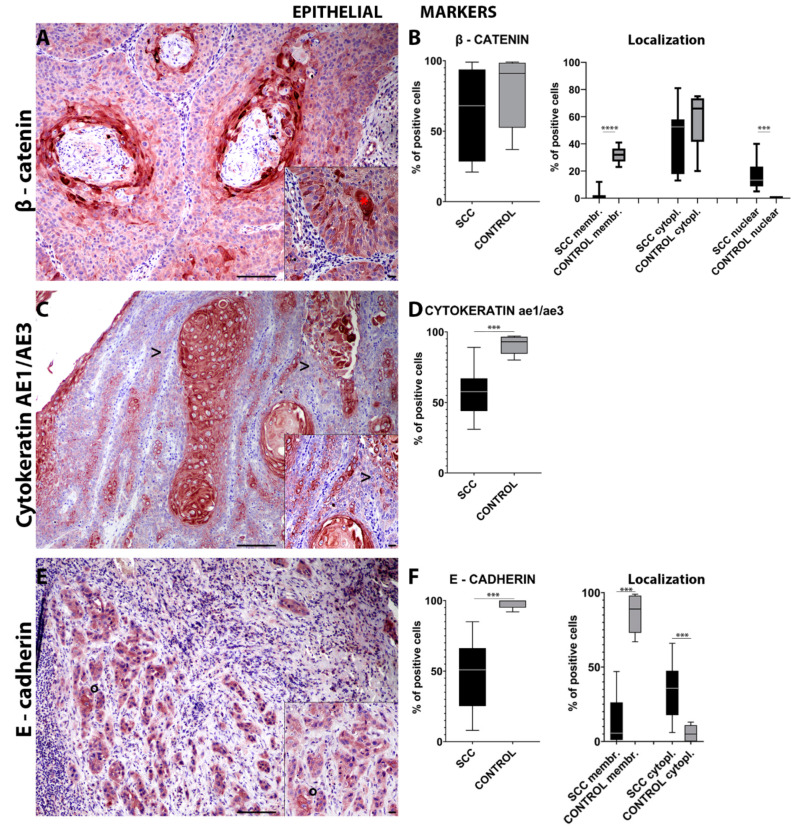
Immunohistochemistry for β-catenin (**A**) in the invasive front of equine penile squamous cell carcinoma (epSCC) shows cytoplasmic and nuclear immunolabeling of the tumor cells (*—(**A**) insert). Box-and-whisker plots show a slightly lower number of cells immunolabeled for β-catenin in the epSCC samples (**B**). Box-and-whisker plots showing control tissue with a higher number of cells expressing membranous β-catenin, control tissue with a higher number of cells expressing cytoplasmic β-catenin, epSCC with a higher number of cells with nuclear β-catenin expression (**B**). Immunohistochemistry for cytokeratin AE1/AE3 (**C**) shows a frequent lack of expression in the cells on the epSCC invasive front (>—(**C**) insert). Box-and-whisker plots show a lower number of cell immunolabeled for cytokeratin AE1/AE3 in the epSCC samples (**D**). Box-and-whisker plots showing control tissues with a higher number of cells expressing cytoplasmic cytokeratin AE1/AE3 (**D**). Immunohistochemistry for E-cadherin (**E**) shows an occasional membranous expression in the cells on the epSCC invasive front (°—(**E**) insert). Box-and-whisker plots show a lower number of cells immunolabeled for E-cadherin in the epSCC samples (**F**). Box-and-whisker plots showing control tissues with a higher number of cells expressing membranous E-cadherin and epSCC samples with a higher number of cells expressing cytoplasmic E-cadherin (**F**). Scale bar = 100 µm and scale bar-insert = 20 µm. All graphs represent box-and-whisker plots with *** *p* ≤ 0.001 and **** *p* ≤ 0.0001. SCC: squamous cell carcinoma group; SCC cytopl: squamous cell carcinoma group cytoplasmic; SCC membr: squamous cell carcinoma group membranous.

Beta (β)-catenin immunohistochemical analysis showed a lack of statistical significance in the number of immuno-positive cells in the invasive front of the tumor compared with the control tissues (Figure 1A). Interestingly, epSCC samples showed a significant (*p* = 0.0002) higher number of cells with nuclear β-catenin expression compared to normal mucosa. On the other hand, the control tissues displayed a significant (*p* < 0.0001) higher number of cells expressing membranous β-catenin. However, cytoplasmic β-catenin localization did not show a statistically significant difference (Figure 1B).

**Figure 2:**
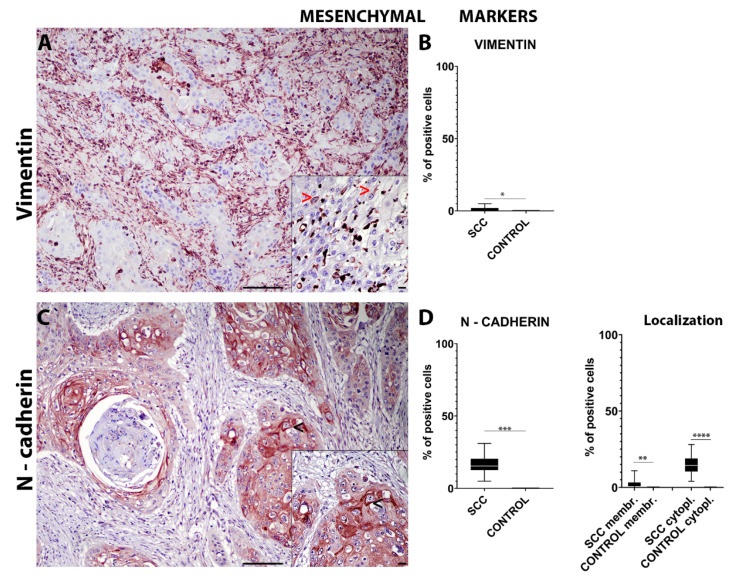
Immunohistochemistry for vimentin (**A**) shows an occasional expression in the cells on the epSCC invasive front (>—(**A**) insert). Box-and-whisker plots show a higher number of cells immunolabeled for vimentin in the epSCC samples (**B**). Box-and-whisker plots showing a higher number of cells expressing cytoplasmic vimentin in the epSCC samples (**B**). Immunohistochemistry for N-cadherin (**C**) often shows a membranous expression in the cells on the epSCC invasive front (<—(**C**) insert). Box-and-whisker plots show a higher number of cells immunolabeled for N-cadherin in the epSCC samples (**D**). Box-and-whisker plots showing a higher number of cells expressing membranous and cytoplasmic N-cadherin in the epSCC samples (**D**). Scale bar = 100 µm and scale bar-insert = 20 µm. All graphs represent box-and-whisker plots with * *p* < 0.05, ** *p* ≤ 0.01, *** *p* ≤ 0.001, and **** *p* ≤ 0.0001. SCC: squamous cell carcinoma group; SCC cytopl: squamous cell carcinoma group cytoplasmic; SCC membr: squamous cell carcinoma group membranous.

Immunohistochemical analysis for intermediate filaments such as cytokeratins (pan-cytokeratin AE3/AE1) revealed a significant (*p* = 0.0004) lower number of cells expressing cytoplasmic cytokeratins in the invasive front of the tumor when compared to the control tissues (Figure 1C,D). On the other hand, the number of cells expressing cytoplasmic vimentin in the invasive front of the tumor was significantly (*p* = 0.0494) higher compared to that in normal mucosa (Figure 2A,B).

Lastly, immunohistochemical analysis of the samples for adhesion molecules such as E-cadherin revealed a significant (*p* = 0.0002) overall lower number of cells expressing E-cadherin in the invasive front of the tumor when compared to the control tissues (Figure 1E). Interestingly, epSCC samples showed a significant (*p* = 0.0008) higher number of cells with a cytoplasmic E-cadherin expression compared to normal mucosa. The E-cadherin membranous expression was significantly (*p* = 0.0002) higher in the normal mucosa compared to the epSCC (Figure 1F). However, the overall number of cells expressing N-cadherin was found to be significantly (*p* = 0.0002) higher in the invasive front of the tumor compared to the control tissues (Figure 2C). In addition, epSCC samples showed a significant higher number of cells both with a membranous (*p* = 0.0083) and a cytoplasmic (*p* < 0.0001) N-cadherin expression compared to the normal mucosa (Figure 2D).

Taken together, intermediate filaments rearrangements from cytokeratins to vimentin and cadherin switching from E-cadherin to N-cadherin in the invasive front of the tumors were highly suggestive of an EMT process in EcPV-2-associated epSCC. Moreover, the increased expression of nuclear β-catenin, known to interact with EMT transcription factors [12,21], suggested to investigate further on the main EMT transcription factors such as TWIST-1 and ZEB-1.

### 2.3. TWIST-1 Nuclear Expression Is Found in EcPV2-Associated Equine Penile Squamous Cell Carcinomas Undergoing the EMT Process

Given that the previous findings were highly suggestive of an EMT process in EcPV-2-associated epSCC and that the increased nuclear β-catenin expression led to a further investigation on the main EMT transcription factors, an immunohistochemical analysis for TWIST-1 and ZEB-1 was performed.

**Figure 3:**
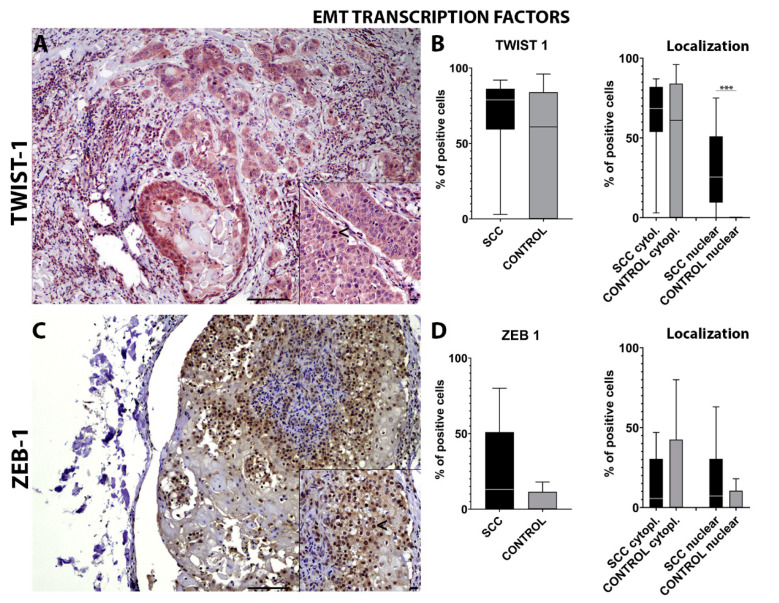
Immunohistochemistry for TWIST-1 (**A**) shows an occasional nuclear and cytoplasmic expression in the cells on the epSCC invasive front (<—(**A**) insert). Box-and-whisker plots show a higher number of cells immunolabeled for TWIST-1 in the epSCC samples (**B**). Box-and-whisker plots showing a higher number of cells expressing cytoplasmic and nuclear TWIST-1 in the epSCC samples (**B**). Immunohistochemistry for ZEB-1 (**C**) shows nuclear expression in the cells on the epSCC invasive front (<—(**C**) insert). Box-and-whisker plots show a higher number of cells immunolabeled for ZEB-1 in the epSCC samples (**D**). Box-and-whisker plots showing control tissue with a higher number of cells expressing cytoplasmic ZEB-1 and epSCC with a higher number of cells with nuclear ZEB-1 expression (**D**). Scale bar = 100 µm and scale bar-insert = 20 µm. All graphs represent box-and-whisker plots with *** *p* ≤ 0.001. SCC: squamous cell carcinoma group; SCC cytopl: squamous cell carcinoma group cytoplasmic.

TWIST-1 immunohistochemical analysis showed a lack of statistical significance in the number of positive cells on the invasive front of the tumor compared with the control tissues (Figure 3A). Interestingly, epSCC samples showed a significant (*p* = 0.0009) higher number of cells with a nuclear TWIST-1 expression compared to the normal mucosa. No statistically significant differences were detected for the cytoplasmic expression of TWIST-1 (Figure 3B).

ZEB-1 immunohistochemical analysis showed a lack of statistical significance in the number of positive cells on the invasive front of the tumor compared with the control tissues (Figure 3C). No statistically significant differences were found for nuclear and cytoplasmic ZEB-1 expression (Figure 3D).

Taken together, the significantly higher number of cells in the invasive front of the tumor with TWIST-1 nuclear immunolabelling might confirm the EMT process in EcPV-2-associated epSCC. Moreover, based on our previous findings [6] and on the increased number of cells with nuclear rather than membranous β-catenin expression, we wanted to further investigate the mRNA levels of genes related to the wnt/β-catenin pathway and to the EMT process, such as *RANKL*, *BCATN1*, *LEF1*, *FOSL1*.

### 2.4. The wnt/β-Catenin Pathway Is Activated in EcPV2-Associated Equine Penile Squamous Cell Carcinomas Undergoing the EMT Process

In order to verify whether EcPV2-associated epSCC were undergoing the EMT process also by activating the canonical wnt/β-catenin pathway, the mRNA levels of *RANKL*, *BCATN1*, *LEF1*, and *FOSL1* were analyzed.

**Figure 4:**
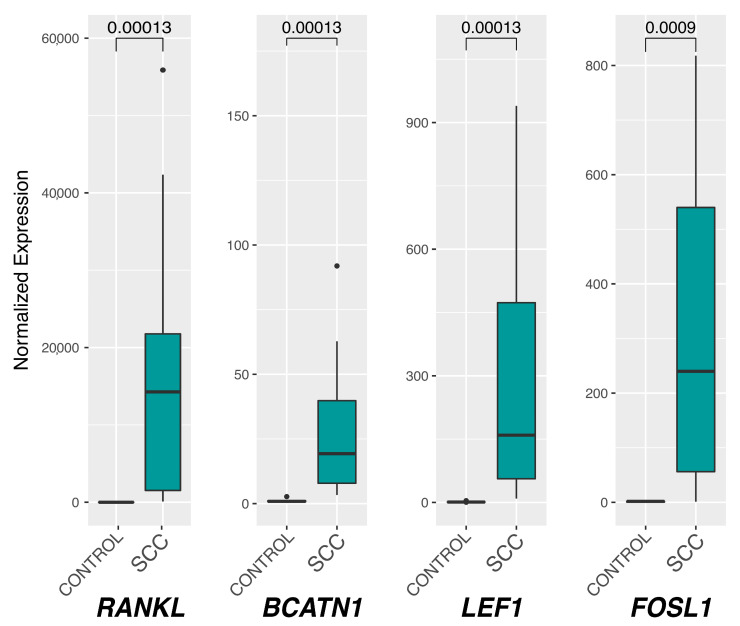
Box plot of *RANKL*, *BCATN1*, *LEF1*, and *FOSL1* relative gene expression. The Mann–Whitney U test was used to assess the median differences between Control and SCC groups and their statistical significance (*p* < 0.05, reported on the upper part of the graphs) after normalizing the expression levels with respect to those of the reference *B2M*. SCC: squamous cell carcinoma group.

All the investigated genes showed a statistically significant increase of the expression in epSCC samples (SCC group) compared to the controls (Control group). In particular, *RANKL* showed a remarkable increase in SCC samples compared to the controls, with relative expression medians of 0.72 for the control group and 14,263.10 for SCC (*p* = 0.0001). Regarding *BCATN1*, the control median was 0.88, while it was 19.32 (*p* = 0.0001) for the SCC group; *LEF1* medians were 0.84 for the control and 159.34 for SCC (*p* = 0.0001), while *FOSL1* showed medians of 1.81 and 239.85 (*p* = 0.0009) for control and SCC groups, respectively (Figure 4).

Taken together, the significant increased mRNA levels of *RANKL*, *BCATN1*, *LEF1*, and *FOSL1* genes in EcPV2-associated epSCC compared to healthy controls might indicate that these tumors undergo EMT also through the activation of the wnt/β-catenin pathway.

## 3. Discussion

Epithelial–mesenchymal transition (EMT) is a highly coordinated sequential biological process, in which, after the activation of “master genes Regulators” (*TWIST*, *ZEB*, *SNAIL1*, SLUG), epithelial cells lose their distinctive characteristics and assume a mesenchymal phenotype [13]. Cells undergo a molecular “reprogramming” determined by the downregulation of epithelial biomarkers and the upregulation of mesenchymal biomarkers. The distinctive feature of the activation of the EMT process is the sub-regulation of the E-cadherin adhesion molecule which, through the process of “cadherin switching”, is replaced by N-cadherin, that allows the cell to acquire migratory–invasive capabilities [12,22]. The sub-regulation of E-cadherin determines the cytoplasmic accumulation and nuclear translocation of β-catenin, which can act as an EMT inducer. Epithelial cells subjected to EMT lose cell–cell adhesion molecules, modulate their polarity, and rearrange their cytoskeleton, which becomes dynamic and flexible following the replacement of cytokeratin by vimentin [12,22].

The immunohistochemical panel used in the current study revealed an overall decreased number of cells expressing epithelial markers at the invasive front of the tumor together with an increased number of cells expressing mesenchymal markers and key EMT transcription factors. Specifically, a lower number of cells expressing E-cadherin and cytokeratin were detected together with an increased number of cells expressing N-cadherin and vimentin, suggesting an EMT process. These findings were further supported by a significantly increased number of cells expressing nuclear TWIST-1 and β-catenin.

Interestingly, TWIST-1 is considered among the main regulators of EMT [23,24,25]. This protein is upregulated in a large number of malignant tumors, determining the onset of the metastatic process via promoting invasiveness in both spontaneous and experimental models [23,26]. Noteworthy, the tumors in the present study had both a high number of cells expressing TWIST-1 and a low number of cells expressing membranous E-cadherin. In addition, the tumors exhibited also a significantly higher number of cells with a cytoplasmic immunolabeling for E-cadherin. This aberrant cytoplasmic internalization has been recently related in equine penile carcinoma to a more aggressive behavior due to AKT/MAPK pathway activation [27,28].

In the present study, there was also another interesting protein localization within the neoplastic cells, namely, the nuclear β-catenin immunolabeling. Nuclear localization of β-catenin is essential for the progression of various human cancers via transcriptional upregulation of downstream genes [29]. Taking into consideration that nuclear β-catenin interaction with the EMT transcription factors might be a driving force for EMT in epSCC and considering our previous insights obtained by investigating the RANK/RANKL and the wnt/β-catenin pathways in equine genital squamous cell carcinomas [6], the mRNA levels of genes related to the wnt/β-catenin pathway and to the EMT process, such as *RANKL*, *BCATN1*, *LEF1*, *FOSL1* were analyzed in the epSCC of the current study.

Interestingly, the results of the current study revealed a significant upregulation of *RANKL*, together with *BCATN1*, *LEF1*, and *FOSL1*. These results might suggest a canonical Wnt pathway activation similarly to what is reported in human penile squamous cell carcinomas [30]. Interestingly, PV oncoproteins, in particular E6, was reported in a transgenic mouse model to enhance the nuclear accumulation of β-catenin and the accumulation of cellular β-catenin-responsive genes, promoting the activation of the wnt/β-catenin pathway in the skin epidermis [31]. However, an over-activation of the wnt/β-catenin pathway alone has been employed solely to induce penile SCC in a transgenic mouse model [32]. Therefore, we might speculate that in our study, the over-activation of the wnt/β-catenin pathway might be the result of EcPV2 E6 interaction, but we cannot exclude that the wnt/β-catenin pathway was just activated, regardless of PV oncoproteins, by the neoplastic progression of the penile squamous cell carcinoma itself.

The E-cadherin decreased expression and the increased N-cadherin and vimentin expression reported in the current study, that also define the EMT process in epSCC, might be indeed influenced by nuclear β-catenin expression [33]. In particular, LEF1 is also considered a facilitator of the EMT process [34,35,36,37]. Normally, β-catenin is associated with E-cadherin intracytoplasmic domain. However, in instances of E-cadherin inhibition, free β-catenin localizes in the nucleus where it can bind and activate LEF1, which, in turn, can decrease E-cadherin expression and increase N-cadherin and vimentin expression by binding to their different promoter regions [38,39]. Similarly, nuclear β-catenin expression and increased LEF1 levels are reported in migratory, vimentin-expressing oral squamous cancer cells (OSCC) and breast cancer cells [40,41].

In addition to the wnt/β-catenin pathway, another pathway reported to promote the EMT process when activated, is the RANKL/RANK pathway [42,43]. Binding of the ligand RANKL to the RANK receptor activates a wide range of signaling cascades, including NF-kB [44], and promotes epithelial-to-mesenchymal transition (EMT) [42,43]. Therefore, the significant upregulation of the RANKL mRNA level observed in this study might suggest a possible involvement of the RANK/RANKL pathway in the EMT process in epSCC, as described in humans [45].

Taken together, we might postulate that epSCC are likely to be characterized by the activation of the RANK/RANKL and wnt/β-catenin pathways that, as a consequence, would promote the EMT process characterized by cadherin switching, cytoplasmic intermediate filament rearrangement, and nuclear expression of TWIST-1 and β-catenin.

It is important to remember that the epSCC samples that were further analyzed in the present study, all tested positive for EcPV2. Precisely, 75% expressed EcPV2 E6 oncogene and 68.8% both E6 and E7 oncogenes. Therefore, it is also important to consider the potential role of EcPV2 oncogenes in triggering the EMT process. Interestingly E6 and E7 oncogenes enhance the expression of TWIST-1 in non-small cell lung cancer (NSCLC) cells, and E7 expression has been correlated with E-cadherin, N-cadherin, and TGF-β expression [46], thus promoting the EMT process [47].

In conclusion, this study reported for the first time in the equine specie a wide immunohistochemical characterization of the EMT process in epSCC, characterized by a decreased number of cells expressing epithelial markers (E-cadherin and cytokeratin) and an increased numbers of cell expressing mesenchymal markers (N-cadherin and vimentin) and EMT-related transcription factors such as TWIST-1. Moreover, this study reported also that epSCC are characterized by the upregulation of *RANKL*, *BCATN1*, *LEF1*, and *FOSL1* genes, which further corroborate the hypothesis of an EMT process.

In conclusion, the authors believe that the numerous similarities of the presented equine SCC model with the human disease and the EMT process occurring in human patients with penile SCC represent a major point of strength of the current study. Indeed, the high reliability of this model based on spontaneous equine lesions has been already pointed out in the last years [7,9]. It is noteworthy to underline how human penile SCC has been reported to undergo the EMT process [48], in particular displaying loss of E-cadherin expression and over-expression of vimentin [49], in line with the results of the current study. Moreover, the epSCC examined in the present study were characterized by the activation of some pathways reported also in human penile SCC or in murine models of penile SCC, namely, the wnt/β-catenin pathway [30,31,32] and the RANK/RANKL pathway [45].

However, future study is warranted to fill one limitation of this study, namely, the lack of a relevantly large group of epSCC not expressing the E6/E7 oncogenes, in order to assess more precisely which role PV oncogenes play in favoring pathways such as wnt/β-catenin or the EMT process in these tumors.

According to the authors, the present work establishes a promising starting point for further investigation about EcPV oncogenes expression and the EMT-related transcription factors and structural/adhesion proteins in EcPV2-associated epSCC. This study provides results to promote future studies in the equine species as a promising spontaneous animal model for human genital squamous cell carcinomas undergoing the EMT process. Therefore, exploiting the future use of the equine model might allow increasing our knowledge in this field and provide a so far unexploited basis for the development of novel targeted therapies inhibiting the EMT process in metastatic human penile SCC.

## 4. Materials and Methods

### 4.1. Samples

Eighteen cases of horse penile cancer were selected and included in the squamous cell carcinoma group (SCC). Penile cancers were sampled during the therapeutic intervention performed to remove the tumor. Each surgery was performed at the equine teaching hospital of Perugia University. The owners provided informed consent to use the sample. Inclusion criteria were: availability of >0.5 cm^2^ of formalin-fixed, paraffin-embedded (FFPE) tissue and histological diagnosis of SCC. A total of 5 normal penises, obtained from the slaughterhouse, were used as the healthy, EcPV2-free, control group (Control). Samples were processed according to the same protocol followed for the tumor tissues. After EcPV2 DNA assessment and histopathological analysis, given that two animals resulted negative for EcPV2 DNA and one sample resulted to be a carcinoma in situ (CIS), the sample size that went through further investigation was reduced to 15 animals.

### 4.2. Histopathological Diagnosis and Immunohistochemistry

Histopathological diagnosis was confirmed on routinely stained slides (hematoxylin and eosin) by two boarded certified pathologists (IP and CB).

The primary antibodies E-cadherin, β-catenin, ZEB-1, vimentin, TWIST-1, N-cadherin, and pan-cytokeratin AE1/AE3, after being titrated according to the manufacturer’s recommendations and being verified to cross-react with the equine species [19], were employed for immunohistochemistry, as previously described [19]. Briefly, after dewaxing–rehydration, tissue sections were exposed to antigen retrieval. Sections were cooled at room temperature for 20 min and soaked into 3% H_2_O_2_ for 12 min. Incubation with primary antibody was carried out overnight at 4 °C. After this, the slides were incubated for 30 min with a biotinylated secondary antibody. Afterwards, an avidin–biotin complex (ABC) peroxidase kit (Vectastain, Elite, ABC-Kit PK-6100, Vector Labs, Burlingame, CA, USA) and a 3′3′-diaminobenzidine (DAB) system (DAB-Kit-SK4100, Vector Labs, Burlingame, CA, USA) were used for the detection of positive reactions. Nuclei were counterstained with Mayer’s hematoxylin. Antibody details are reported in Table 2. For negative controls, specific primary antibodies were replaced by ascitic fluid from non-immunized BALB/cJ mice (for E-cadherin, vimentin, and pan-cytokeratin AE1/AE3), serum from non-immunized rabbits (for N-cadherin, TWIST-1, and ZEB-1), and serum from non-immunized goats (for β-catenin). The dilution of the negative controls was chosen according to the protein concentration of the replaced primary antibodies. EMT markers expression was assessed manually by counting 10 evenly distributed fields within the tumor periphery/invasive front at 400x magnification using a Nikon Eclipse E800 microscope (Nikon Corporation, Japan) with a Nikon PLAN APO lens and equipped with a Camera DIGITAL SIGHT DS-Fi1 (Nikon Corporation, Tokyo, Japan). Pictures were acquired with a DS camera control unit DS-L2 (Nikon Corporation, Japan) and stored in a USB device. For control samples, 10 high-power fields (400×) evenly distributed within the mucosal epithelial layer were analyzed.

### 4.3. DNA Extraction and EcPV2 Detection

DNA extraction was performed from FFPE samples as previously described [6]. DNA concentration was evaluated by QUBIT 3 (ThermoFisher, Waltham, MA, USA), and 100 ng of DNA was used to test EcPV2 E7 and E6 presence. Moreover, Beta-2-microglobulin (*B2M*) gene was applied to assess DNA amplifiability. Primer and probe sequences are reported in Table 3. Real-time PCR was performed as previously described [50]. Briefly, 200 nM of the probe and 100 nM of each primer were added to the TaqDNA Polymerase MasterMix (Biorad, Hercules, CA, USA). Amplification was performed into in a CFX96™ Real-Time System, and a Cq of 38 was set as cut-off for virus positivity.

### 4.4. RNA Extraction and EcPV2 Gene Expression

Total RNA was extracted from five sections (5 µm) of each FFPE sample using RecoverAll™ Total Nucleic Acid Isolation Kit for FFPE (Invitrogen™) according to the manufacturer’s instructions. Gene expression of *E6* and *E7* EcPV2 was tested using specific probes and primers sets (Table 3). Two hundred-fifty ng of RNA was used for reverse transcription (RT) that was performed using the SuperScript™ IV VILO™ Master Mix (Invitrogen, ThermoFisher Scientific, Waltham, MA, USA). Real-time PCR was performed as described above (see Section 4.3). RNA was used as a control to exclude possible contaminations by EcPV2 genomic DNA.

### 4.5. RT-qPCR for Host Gene Study

In this study, receptor activator of nuclear factor-kappa B ligand (*RANKL*), β-catenin (*BCATN1*), FOS like 1 (*FOSL1*), and lymphoid enhancer binding factor 1 (*LEF1*) were selected for gene expression evaluation; the respective primer sets are reported in Table 4. *B2M* was chosen as a reference gene as previously described [6], and a real-time PCR amplification using the Power SYBR™ Green PCR Master Mix (Applied Biosystems, Thermo Fisher Scientific, Waltham, MA, USA) was performed in a CFX96™ Real-Time System with 5 μL of 1:5 diluted cDNA [51]. Each sample was tested in triplicate, and fluorescence data were collected at the end of the second step of each cycle. Relative expression was calculated through the 2^−ΔΔCq^ method. For samples with no detectable amplification, a Cq of 42 was chosen as the detection threshold.

### 4.6. Statistical Analysis

Immunohistochemical results for the investigated markers were evaluated for normality distribution with the Shapiro–Wilk test, followed by the Manny-Whitney test. Statistical significance for each analysis was set at a *p*-value ≤ 0.05. All statistical analyses were performed with GraphPad Prism version 8.0.1 for Windows (GraphPad Software, La Jolla, CA, USA). Gene expression values were checked for normality distribution through the Shapiro–Wilk test, and non-parametric tests were chosen to test the hypothesis. The Mann–Whitney U test was performed to assess differences in gene expression between Control (C) and Tumor (T) groups, while the Kruskal–Wallis H test was used to evaluate median differences between C, E6/E7+, and E6/E7− samples, applying a Mann–Whitney U test for pairwise comparison. The *p*-value (P) threshold was set at 0.05 for statistical significance. Differential expression was performed in R (v. 4.0.3, R Foundation for Statistical Computing, Vienna, Austria).

## Figures and Tables

**Table 1:** Histological diagnosis, koilocytosis, EcPV2-E6 detection, and *E6*/*E7* gene expression.

Case ID	Histological Diagnosis	Koilocytosis	DNA		cDNA		Grouping on the Expression of Viral Genes
			E7	E6	E6	E7	E6/E7
1	SCC	N	+	+	30.4 ± 0.2	30.4 ± 0.3	E6/E7+
2	SCC	N	+	+	36.2 ± 0.6	35.8 ± 0.5	E6/E7+
3	SCC	N	+	+	>48	>48	E6/E7−
4	SCC	N	+	+	33.5 ± 0.4	37.2 ± 0.6	E6/E7+
5	SCC	N	+	+	32.7 ± 0.9	32.2 ± 0.3	E6/E7+
6	SCC	N	+	+	33.5 ± 0.3	37.8 ± 0.5	E6/E7+
7	SCC	N	+	+	>48	>48	E6/E7−
8	SCC	N	+	+	33.7 ± 0.4	32.6 ± 0.2	E6/E7+
9	SCC	N	+	+	>48	>48	E6/E7−
10	SCC	N	+	+	>48	>48	E6/E7−
11	SCC	N	+	+	33.9 ± 1.9	32.6 ± 0.3	E6/E7+
12	SCC	N	-	-	ND	ND	E6/E7−
13	CIS	N	+	+	30.4 ± 0.6	29.4 ± 0.5	E6/E7+
14	SCC	N	+	+	32.3 ± 0.1	>48	E6/E7+
15	SCC	N	-	-	ND	ND	E6/E7−
16	SCC	Y	+	+	23.4 ± 0.1	21.6 ± 0.4	E6/E7+
17	SCC	N	+	+	21.2 ± 0.6	22.4 ± 0.7	E6/E7+
18	SCC	N	+	+	22.9 ± 0.2	21.4 ± 0.3	E6/E7+

CIS: carcinoma in situ; N: no; ND: not detectable; SCC: squamous cell carcinoma; Y: yes.

**Table 2:** Details of the antibodies used for immunolabelling, including primary antibody, host species, clonality, epitope retrieval method, dilution of primary antibody, secondary antibody, and positive control.

Target Antigen	Antibody Details/Clone	Heat Induced Epitope Retrieval (HIER)	Primary Antibody Dilution	Secondary Antibody (1:200)	Positive Control
E-cadherin	Monoclonal mouse anti-human, IgG2a, clone 36/E-Cadherin BD 610181 (BD transduction laboratories, Franklin lakes, NJ, USA)	Microwave 400 W, 3 cycles, 5 min. each, sodium citrate buffer, pH 6.0	1:100	Biotinylated goat anti-mouse IgG (BA-100—Vector Labs)	Horse, skin
Pan- cytokeratin AE1/AE3	Monoclonal mouse anti-human IgG1, SC-81714 (Santa Cruz Biotechnology, Dallas, TX, USA)	Microwave 400 W, 3 cycles, 5 min. each, sodium citrate buffer, pH 6.0	1:100	Biotinylated goat anti-mouse IgG (BA-100—Vector Labs)	Horse, skin
β-catenin	Polyclonal goat anti-human IgG, AB0095-200 (Sicgen, Coimbra, Portugal)	Microwave 400 W, 3 cycles, 5 min. each, sodium citrate buffer, pH 6.0	1:3000	Biotinylated rabbit anti-goat IgG (BA-100—Vector Labs)	Horse, intestine
N-cadherin	Polyclonal rabbit anti-human IgG, 22018-1-AP (proteintech, Rosemont, IL, USA)	Microwave 400 W, 3 cycles, 5 min. each, sodium citrate buffer, pH 6.0	1:3000	Biotinylated goat anti-rabbit IgG (BA-100—Vector Labs)	Horse, heart
Vimentin	Monoclonal mouse anti-human IgG1, Clone RV202 SC-32322 (Santa Cruz Biotechnology, Dallas, TX, USA)	Microwave 400 W, 3 cycles, 5 min. each, sodium citrate buffer, pH 6.0	1:100	Biotinylated goat anti-mouse IgG (BA-100—Vector Labs)	Horse, heart (endothelial cells)
ZEB-1	Polyclonal rabbit anti-human IgG, LS-C31478 (LSBio, Seattle, WA, USA)	Microwave 400 W, 3 cycles, 5 min. each, sodium citrate buffer, pH 6.0	1:200	Biotinylated goat anti-rabbit IgG (BA-100—Vector Labs)	Horse, kidney
TWIST-1	Polyclonal rabbit anti-human IgG, orb-329955 (biorbyt, Cambridge, UK)	Microwave 400 W, 3 cycles, 5 min. each, sodium citrate buffer, pH 6.0	1:800	Biotinylated goat anti-rabbit IgG (BA-100—Vector Labs)	Horse, kidney

**Table 3:** Probes and primers sets used to evaluate EcPV2 presence and expression.

Gene	Sequences	Reference/Accession
EcPV2-E7	F-5′-CTCTGAGCAGCATCACCCTT-3′ R-5′-TCTTCCTCGTCTTCTGTGTCC-3′	NC_012123
p-EcPV2-E7	FAM-AGAGCGCTCCCCCTCAGTCA-TAMRA	NC_012123
EcPV2-E6	F-5′-CGTTGGCCTTCTTTGCATCT-3′ R-5′-AGGTTCAGGTCTGCTGTGTT-3′	[7]
p-EcPV2-E6	FAM-CCGTGTGGCTATGCTGATGACATTTGG-TAMRA	[7]
B2M DNA detection	F-5′-CTGATGTTCTCCAGGTGTTCC-3′ R-5′-TCAATCTCAGGCGGATGGAA-3′	[50]
B2M cDNA expression	F-5′-GGCTACTCTCCCTGACTGG-3′ R-5′-TCAATCTCAGGCGGATGGAA-3′	[50]
p-B2M	FAM-ACTCACGTCACCCAGCAGAGA-TAMRA	[50]

**Table 4:** Primers Set for gene expression.

Gene	Primer Pairs Sequences	Reference
*B2M*	F-5′-GGCTACTCTCCCTGACTGG-3′ R-5′-TCAATCTCAGGCGGATGGAA-3′	[50]
*RANKL*	F-5′-AGCCTGACACTCAACCTTTTG-3′ R-5′-CCAGGAAGACAGACTCACTTTG-3′	[6]
*BCATN1*	F-5′-CCTCTTCAGAACGGAGCCAA-3′ R-5′-CTGGCGATATCCAAGGGGTT-3′	[6]
*FOSL1*	F-5′-TACCGAGACTTCGGGGAAC-3′ R-5′-GCGTTGATACTTGGCACGAG-3′	[6]
*LEF1*	F-5′-GCCAGACAAGCACAAACCTC-3′ R-5′-GGGTCCCTTGCTGTAGAGG-3′	[6]

## Data Availability

Not applicable.
